# Biological sex differences in pharmacokinetics and adverse drug reactions

**DOI:** 10.1007/s00210-025-04721-8

**Published:** 2025-10-21

**Authors:** Ahmad Aljohmani, Daniela Yildiz

**Affiliations:** 1 Molecular Pharmacology, Center for Molecular Signaling (PZMS), Saarland University, Homburg, Saar, Germany; 2 Center for Human and Molecular Biology (ZHMB), Saarland University, Homburg, Saar, Germany; 3 Pharma Science Hub (PSH), Center for Gender-Specific Biology and Medicine (CGBM), Center for Biophysics (ZBP), Saarland University, Saarbrücken, Germany

**Keywords:** Biological sex, Pharmacokinetics, Absorption, Distribution, Metabolism, Excretion

## Abstract

Although physiological and hormonal differences between males and females can significantly alter drug absorption, distribution, metabolism, and excretion (ADME), most current pharmacotherapeutic guidelines remain sex neutral. The chapter aims to elucidate biological sex-specific factors across all pharmacokinetic (PK) phases, subsequent therapeutic efficacy, and adverse drug reactions (ADRs), and to highlight implications for individualized therapy including within-woman variation across the menstrual cycle and other hormonal states. This work is based on an extensive review of current literature and clinical data examining sex-specific variations across all phases of pharmacokinetics. Significant biological sex-related differences were found among all PK phases. Females tend to have slower gastric emptying, higher body fat composition, and lower glomerular filtration rates. CYP3A4 is typically more active in females, while CYP1A2, CYP2D6, and CYP2E1 show greater activity in males. In females, endogenous or exogenous hormonal fluctuation can slightly affect gastric motility, protein binding, and selected CYP activities altering clearance for susceptible drugs. In addition, pregnancy increases plasma volume and glomerular filtration with predictable effects on renally cleared drugs and selected hepatic pathways. These differences influence drug half-life, systemic exposure, and the risk of ADRs. Biological sex is a significant determinant of drug pharmacokinetics. Integrating biological sex-specific data into clinical guidelines is essential to optimize drug efficacy and minimize ADRs. Future research and regulatory action should promote biological sex aware pharmacological practices, encouraging routine inclusion of biological sex analyses in clinical trials.

**Graphical Abstract:**

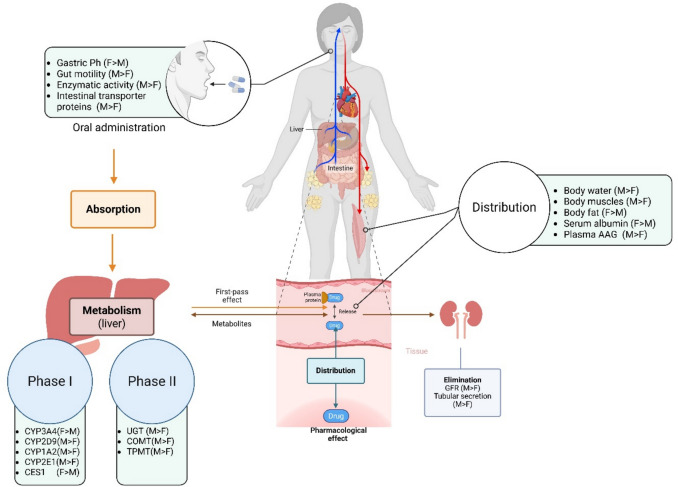

## Introduction

The pharmacokinetics of drugs represent a major pre-step to reflect drug response and the subsequent adverse drug reactions (ADRs). The relationship between dose effect is more vulnerable to influencing factors and harder to predict than the concentration-effect relationship. In fact, a drug undergoes multiple dynamic factors that affect the final optimal therapeutic concentration that reaches the circulation. Drug pharmacokinetics vary based on the physiological status, which vice versa is influenced by the biological sex and the concomitant wide variation in hormonal secretion and anatomical condition. These variations include differences in gastrointestinal (GI) motility, hepatic enzyme activity, fat-to-lean body ratio, plasma protein binding, and renal clearance (Kasarinaite et al. 2023; Gerges and El-Kadi 2023; Shen and Shi 2015). Thereby, sex differences affect drug pharmacokinetics, resulting in under or over therapeutic levels, predisposing to less efficacy or higher ADRs (Li et al. 2019; Estes 1998; O’Connell 1995; Cheymol 2000; Strandell and Wahlin 2011; Johnell and Klarin 2007; Romanescu et al. 2022; LeGates et al. 2019; Keers and Aitchison 2010).

Despite the clear biological sex differences that can affect drug behavior, most pharmacotherapy guidelines do not consider these differences in drug dosing regimens (Beery and Zucker 2011; Hayes and Redberg 2008). In fact, most of the clinical studies were historically male based, assuming similar outcome between both sexes. Currently, sex-specific pharmacokinetic data remain missing for a substantial number of approved drugs, and only a few drugs are prescribed with labeled sex-based dosage. Desmopressin, used to treat nocturia (Juul et al. 2011; Nocdurna 2018), Suvorexant, used to treat insomnia (BELSOMRA 2014), and zolpidem (only in USA), used to treat insomnia (Greenblatt et al. 2019; FDA 2013) are examples of drugs that were changed to sex-specific dosage due to post-marketing ADR reports. Many clinical trials fail to report sex-based PK analyses, while other data often do not differentiate between male and female responses. These disparities highlight the importance of considering biological sex as a critical factor in drug dosing and safety assessments.

## Biological sex difference on pharmacokinetics: an overview

Pharmacokinetics (PK) is a core area of pharmacological science that examines the drugs pathway through the body, ranging from its liberation, absorption, distribution, metabolism, until excretion. These processes, collectively referred to as LADME, are critical to establish the balance between drug efficacy and safety, to determine appropriate dosing. Each phase is interdependent and influenced by various physiological and biochemical factors, that are affected by biological sex (Chaira et al. 2023; Doogue and Polasek 2013; Eddershaw et al. 2000).

The first step in pharmacokinetics is the liberation of the active ingredient from its formulation, and influenced by factors such as drug formulation, gastric pH, and gastrointestinal motility (Koziolek et al. 2019; Caldwell et al. 1995). Absorption refers to the transfer of the drug from the site of administration into systemic circulation. This process is affected by membrane permeability, solubility, and gastrointestinal conditions, including variations in motility and pH (Benedetti et al. 2009). Once absorbed, the drug undergoes distribution, in which it is transported to various tissues and organs. Distribution is affected by physiological parameters such as fat and muscle mass, total body water, blood flow, and protein binding. Biological sex plays a core role in this phase, as differences in body composition, fat distribution, and plasma protein concentrations can change the extent and the rate of drugs disposition (Caldwell et al. 1995; Onetto and Sharif 2025). Metabolism, a key determinant of drug clearance, primarily occurs in the liver through enzymatic modifications that facilitate drug elimination. Drug metabolism is classified into phase I reactions, which involve oxidation, reduction, or hydrolysis, and phase II reactions, which include conjugation processes that enhance drug solubility (Li et al. 2019; Benedetti et al. 2009). The enzymatic activity varies between biological sexes, with CYP3A4 activity being higher in females, often resulting in faster clearance of drugs metabolized by this enzyme, while CYP1A2 and CYP2E1 exhibit greater activity in males (Kasarinaite et al. 2023; Gerges and El-Kadi 2023; Shen and Shi 2015). The final phase of pharmacokinetics process is excretion, the process by which drugs and their metabolites (mediated by the hepatic metabolism) are eliminated from the body, primarily through renal pathway. Males typically exhibit higher glomerular filtration rates (GFR), contributing to faster renal clearance of certain drugs, whereas females often experience reduced renal elimination, leading to prolonged drug half-lives and higher systemic exposure (Caldwell et al. 1995).

### Biological sex differences in drug liberation and absorption

Drug absorption is controlled by physiological and biochemical parameters. While these differences may sometimes seem subtle, variations in gastric pH, gut motility, gut enzymatic activity, and transporter protein expression can contribute to sex-based differences in drug absorption, affecting how efficiently a drug reaches its target site (see Table 1).

**Table 1:** Summary of observed biological sex differences in drug absorption

Parameter	Biological sex difference	Example	Reference
Gastric pH	F > M (F higher)	-	Feldman and Barnett 1991; Soldin and Mattison 2009; Fletcher et al. 1994; Kimura and Higaki 2002; Fleisher et al. 1999)
Gastric emptying	M > F (M faster)	Metoprolol, verapamil, theophylline	Datz et al. 1987; Knight et al. 1997; Stillhart et al. 2020; Eugene 2016; Dadashzadeh et al. 2006; Nafziger and Bertino 1989)
Gut motility and transit time	M > F (M faster)	-	Soldin and Mattison 2009; Fletcher et al. 1994; Kimura and Higaki 2002; Fleisher et al. 1999; Nicolas et al. 2009; Caballeria et al. 1989; Stephen et al. 1986)
GI enzymes activity	M > F (M higher)	alcohol	Parlesak et al. 2002; Baraona et al. 2001)
Transporter Proteins	M > F (M higher)	-	Nicolas et al. 2009; Englund et al. 2006; Mai et al. 2021)

#### Gastric pH, gut motility, and enzymatic activity

Fasting gastric pH has been reported to be higher in females ((2.79 ± 0.18) compared to males (2.16 ± 0.09) (Feldman and Barnett 1991). This reduced gastric pH in males can trigger the ionization of weak bases drugs or the degradation of acid sensitive drugs. The slower gastric emptying observed in females prolongs the residence time of drugs in the stomach, potentially delaying absorption kinetics (e.g., metoprolol, theophylline, and verapamil) (Soldin and Mattison 2009; Fletcher et al. 1994; Kimura and Higaki 2002; Fleisher et al. 1999; Xiang et al. 2024; Datz et al. 1987; Knight et al. 1997; Stillhart et al. 2020; Eugene 2016; Dadashzadeh et al. 2006; Nafziger and Bertino 1989). Gastrointestinal enzymes such as alcohol dehydrogenase (ADH) also exhibit sex-based differences. Higher ADH activity in males leads to faster metabolism of substances such as alcohol, reducing systemic bioavailability compared to females (Parlesak et al. 2002; Baraona et al. 2001). With 91.7 h in females and 44.8 h in males, gut motility and intestinal transit times differ, attributed to hormonal factors such as progesterone, which slows gastrointestinal motility. Thus, prolonged transit times can increase the exposure of drugs to intestinal absorption sites, enhancing the bioavailability of poorly soluble compounds (Nicolas et al. 2009; Caballeria et al. 1989; Stephen et al. 1986). However, for rapidly absorbed drugs, delayed transit time may extend the time to peak plasma concentration. Diltiazem absorption has been shown to be influenced by intestinal transit time. Since females generally exhibit longer gastrointestinal transit times compared to males, it is assumed that this physiological difference may impact the absorption of diltiazem. However, no direct studies have investigated the effect of sex-related differences in intestinal transit time on diltiazem absorption (Zimmermann et al. 1999). Sex differences in bile acid composition and enterohepatic circulation can further modulate drug solubility. Males have higher levels of cholic acid, while females exhibit greater concentrations of chenodeoxycholic acid, altering micellar solubilization and the absorption of lipophilic drugs (Nicolas et al. 2009; Fisher and Yousef 1973; Soldin et al. 2011). For instance, cyclosporine absorption is influenced by bile composition and may vary between males and females due to sex-specific differences in bile acid profiles. However, no direct evidence has been reported to confirm a sex-related difference in cyclosporine absorption (Winkler et al. 1995).

#### Transporter proteins and intestinal dynamics

Transport proteins in the gastrointestinal tract can modulate drug absorption. Efflux transporters like P-glycoprotein (P-gp) actively transport substrates out of enterocytes back into the intestinal lumen, limiting systemic drug exposure. P-gp expression reported to be higher in males, resulting in reduced absorption of its substrates compared to females (Nicolas et al. 2009; Englund et al. 2006; Mai et al. 2021). However, this needs more investigation. Hormonal regulation further complicates this relationship, with estrogen and progesterone shown to modulate the expression of both efflux and uptake transporters, altering drug bioavailability across the menstrual cycle and during pregnancy (Soldin et al. 2011). Table 1 summarizes the observed biological sex differences in drug absorption.

### Drug distribution

Key parameters for drug distribution such as total body water, fat content, and lean body mass vary significantly between biological sexes, affecting the volume of distribution (Vd) and, consequently, drug concentrations in plasma and tissues (see Table 2).

**Table 2:** Summary of observed biological sex differences in drug distribution

Parameter	Biological sex difference	Example	Reference
Total body water	M > F	Salbutamol, ofloxacin, alcohol	Vahl et al. 1997; Arthur et al. 1984; Wedel et al. 1991; Sowinski et al. 1999)
Body fat content	F > M	Diazepam, propofol, vecuronium, rocuronium	Duan et al. 2006; Routledge et al. 1981; Ochs et al. 1981; Greenblatt et al. 1980; Sahinovic et al. 2018; Hoymork and Raeder 2005; Adamus et al. 2008; Semple et al. 1994)
Lean body mass	M > F	Alcohol, fluoroquinolone antibiotics	Soldin et al. 2011; Williams and Leggett 1989; Cunningham 1982; Jones 2010)
Plasma protein binding	Albumin F > M AAG M > F	-	Englund et al. 2006; Weaving et al. 2016; Wiegratz et al. 2003; Walle et al. 1983; Piafsky and Borga 1977; Kishino et al. 1995)
CO and regional blood Flow	M = F (CO) M > F (muscular blood flow) F > M (adipose tissue blood flow)	Vecuronium (muscular blood flow), diazepam, propofol (adipose tissue blood flow)	Soldin and Mattison 2009; Soldin et al. 2011; Sahinovic et al. 2018; Hoymork and Raeder 2005; Semple et al. 1994)

#### Body composition

Males display a higher total body water than females due to lower fat content and higher lean body mass, which can increase the Vd of the water-soluble drugs (Vahl et al. 1997). For the same dose, males exhibit a larger Vd for ethanol (water soluble), leading to lower concentrations and diminished effects compared to females. In contrast, the smaller water compartment in females produces higher peak plasma concentrations (Cmax) and amplifies the effects of hydrophilic drugs (Arthur et al. 1984; Wedel et al. 1991). Additionally, salbutamol (albuterol) and ofloxacin, both depend on distribution in lean body water, exhibit significantly greater Vd values in males (Sowinski et al. 1999).

Females have a markedly higher percentage of body fat compared to males, with fat mass averaging 16.5 kg in females versus 13.5 kg in males. This difference becomes even more pronounced during pregnancy, when fat mass increases to approximately 19.8 kg at full term (Duan et al. 2006). This variation plays a critical role in the distribution of lipophilic drugs, which preferentially partition into adipose tissue. Lipophilic drugs, such as diazepam and propofol, demonstrate higher Vd values in females due to their larger fat stores. The Vd of diazepam in females is reported as 1.87 L/kg compared to 1.34 L/kg in males, leading to prolonged drug half-life and extended sedative effects in women (Routledge et al. 1981; Ochs et al. 1981; Greenblatt et al. 1980; Sahinovic et al. 2018; Hoymork and Raeder 2005). Other lipophilic compounds, including neuromuscular blockers like vecuronium and rocuronium, also show prolonged durations of action in females. The slower redistribution of these drugs from adipose tissue to the circulation delays their clearance and extends their pharmacodynamic effects (Adamus et al. 2008; Semple et al. 1994).

Males have a higher muscle mass in comparison to females which is able to serve as a reservoir for certain drugs providing a greater distribution compartment, particularly the moderately lipophilic or highly ionized drugs (Williams and Leggett 1989; Cunningham 1982). Interestingly, the liver, which is part of lean body mass, represents a larger proportion of total body weight in females. This may partially explain the faster clearance of certain drugs in females, despite lower hepatic blood flow. For instance, alcohol and fluoroquinolone antibiotics are rapidly metabolized in females due to the liver’s enhanced relative capacity, even though systemic alcohol concentrations are initially higher in females owing to their smaller Vd (Soldin et al. 2011; Jones 2010).

#### Plasma protein binding

Plasma protein binding significantly influences the effective drug concentration by regulating the free (active) fraction of drugs in circulation. The primary proteins involved in drug binding are albumin, alpha-1 acid glycoprotein (AAG), and serum-binding globulins (Succari et al. 1990). Sex differences in plasma protein binding are generally limited; however, small difference may affect drugs with high binding affinity and narrow therapeutic windows. Albumin, which primarily binds weakly acidic drugs, shows minimal variation between biological sexes under normal conditions (Succari et al. 1990; Verbeeck et al. 1984). However, an observational study with more than 1 million participants showed a significant increase in females’ serum albumin level compared to males (Weaving et al. 2016). Additionally, AAG, which binds weakly basic drugs, displays lower levels in females due to lower synthesis mediated by estrogen (Englund et al. 2006; Wiegratz et al. 2003; Walle et al. 1983; Piafsky and Borga 1977; Kishino et al. 1995). In addition, endogenous and exogenous estrogens increase the corticosteroid-binding globulin levels in females (Wiegratz et al. 2003). Nevertheless, clinical investigations failed to demonstrate sex-related differences in free fractions of highly bound drugs (e.g., disopyramide, verapamil, and nifedipin) in volunteers receiving hormone replacement therapy or combined oral contraceptives (COCs) (Keefe et al. 1981; Krecic-Shepard et al. 2000a).

#### Regional blood flow

Despite the similar cardiac index (cardiac output (CO) normalized to body surface area) between males and females, the blood flow to different organs and tissues varies between males and females reflecting the differences in body composition (Soldin et al. 2011). Males exhibit higher blood flow to skeletal muscle (17% of CO vs. 12% in females), which facilitates faster drug delivery to muscle tissue (Soldin and Mattison 2009). This difference is particularly relevant for drugs like neuromuscular blockers, whose effects may onset more rapidly in males due to enhanced perfusion. Conversely, females show greater blood flow to adipose tissue (8.5% of CO vs. 5% in males), which prolongs the redistribution and clearance of lipophilic drugs, such as diazepam, leading to longer durations of action (Soldin and Mattison 2009; Gandhi et al. 2004; Whitley and Lindsey 2009).

### Hepatic metabolism

Hepatic metabolism is the primary mechanism by which drugs are chemically modified to facilitate their elimination from the body. This process is divided into two main stages: phase I and phase II reactions. Each phase involves distinct enzymatic pathways that can be regulated by sex-specific differences in activity, hormonal regulation, substrate affinity and concomitant drug administration (see Table 3) (Almazroo et al. 2017; Renton 1986).

**Table 3:** Summary of observed biological sex differences in hepatic metabolism

Enzyme	Biological sex difference	Example	Reference
Phase I			
CYP3A4	F > M	Midazolam, diazepam, nifedipine, verapamil, erythromycin, methylprednisolone	Greenblatt et al. 1980; Krecic-Shepard et al. 2000a; Anderson 2002; Greenblatt and Moltke 2008; Aichhorn et al. 2007; Gorski et al. 1998; Krecic-Shepard et al. 2000b; Watkins et al. 1989; Thummel et al. 1996; Hunt et al. 1992; Lew et al. 1993)
CYP2D6	M > F	Propranolol, clomipramine, nortriptyline	Nicolas et al. 2009; Anderson 2002; Gilmore et al. 1992; Walle et al. 1985; Xie and Chen 1995; Gex-Fabry et al. 1990; Dahl et al. 1996)
CYP1A2	M > F	Caffeine, warfarin, paracetamol, olanzapine, clozapine	Thorn et al. 2012; Grzegorzewski et al. 2021; Wong et al. 2021; Garcia et al. 2005; Whitley et al. 2007; Abernethy et al. 1982a; Callaghan et al. 1999; Lane et al. 1999)
CYP2E1	M > F	Ethanol, chlorzoxazone	Lucas et al. 1995; Kim and O’Shea 1995)
CES1	F > M	Oseltamivir, dabigatran, lovastatin and simvastatin	Shi et al. 2016a; Shi et al. 2016b; Vree et al. 2003; Yang et al. 2014)
Phase II			
UGT	M > F	Propranolol, labetalol, paracetamol	Bock et al. 1994; Schwartz 2003; Walle et al. 1989; Johnson et al. 2000; Allegaert et al. 2015)
COMT	M > F	Levodopa	Boudikova et al. 1990; Floderus et al. 1981; Kumagai et al. 2014; Conti et al. 2022; Martinelli et al. 2003)
TPMT	M > F	6-Mercaptopurine, fluorouracil, doxorubicin	Szumlanski et al. 1992; Klemetsdal et al. 1992; Lennard et al. 1990; Milano et al. 1992; Port et al. 1991; Zalcberg et al. 1998; Dobbs et al. 1995)

#### Phase I reactions

Phase I metabolism encompasses enzymatic modifications such as oxidation, reduction, and hydrolysis. These reactions, primarily mediated by the cytochrome P450 (CYP) family, can increase the polarity of lipophilic drugs, increasing their water-solubility for conjugation in phase II. Lipophilic drugs are particularly susceptible to phase I metabolism due to their ability to cross biological membranes and interact with CYP enzymes. Sex differences in phase I metabolism are particularly evident in CYP enzyme activity, which significantly impacts drug clearance rates and plasma concentrations (Nicolas et al. 2009; Zhou et al. 2005).

- **CYP3A4** is the most abundant human CYP enzyme metabolizing nearly 50% of clinically used drugs. Enhanced CYP3A4 activity in females results in faster clearance of CYP3A4 metabolized drugs such as midazolam, diazepam, nifedipine, verapamil, erythromycin, and methylprednisolone (Greenblatt et al. 1980; Krecic-Shepard et al. 2000a; Anderson 2002; Greenblatt and Moltke 2008; Aichhorn et al. 2007; Gorski et al. 1998; Krecic-Shepard et al. 2000b; Watkins et al. 1989; Thummel et al. 1996; Hunt et al. 1992; Lew et al. 1993), potentially reducing their therapeutic half-live and may necessitate dose adjustment. Thereby, plasma drug concentrations and the therapeutic half-live may be reduced, necessitating dose adjustment (Cunningham 1982; Custodio et al. 2008). The regulation of CYP3A4 activity is influenced by sex-specific growth hormone secretion patterns (Kennedy 2008). Continuous growth hormone release in females enhances CYP3A4 expression, while the pulsatile release observed in males leads to comparatively lower activity (Waxman and Holloway 2009). However, not all CYP3A4 substrates show a similar pattern, and the interpretation of most of the results can be biased by the involved subjects, sample size and hormonal states (Kirkwood et al. 1991; Yee et al. 1984; Holazo et al. 1988; Gupta et al. 1995; Fleishaker and Peters 1996)
- **CYP2D6** metabolizes approximately 25% of all drugs, thereby displaying the second most frequent enzyme involved in therapeutic drug biotransformation. While CYP2D6 activity varies widely due to genetic polymorphisms, slower clearance of its substrates, such as antihistamines, is often observed in females (Nicolas et al. 2009; Anderson 2002). This difference contributes to increased drug exposure and a greater likelihood of ADRs such as sedation. Sex differences in beta-blockers such as propranolol have been shown in Caucasians (Gilmore et al. 1992; Walle et al. 1985) and in Chinese volunteers where propranolol clearance was lower in women compared with men (Xie and Chen 1995). In addition, clomipramine (tricyclic antidepressant) concentrations have been reported to be higher in women compared with men (Gex-Fabry et al. 1990). Moreover, higher nortriptyline concentrations in depressed women were detected compared with men (Dahl et al. 1996). Biological sex may also influence certain prodrug-activation pathways, but effects are often secondary to genotype and drug-drug interactions. For instance, tamoxifen/endoxifen activation depends on CYP2D6, however, biological sex differences are inconsistent and are better explained by CYP2D6 genotype or phenoconversion, adherence, and co-therapies than by biological sex per se (Goetz et al. 2018; Sanchez-Spitman et al. 2019). However, limited data exist regarding general CYP2D6 substrates to generalize the conclusions.
- **CYP1A2** contributes to 13% of total CYP liver proteins (Shimada et al. 1994) with caffeine, clozapine, erythromycin and propranolol as well-documented substrates (Scandlyn et al. 2008; Thorn et al. 2012; Grzegorzewski et al. 2021). CYP1A2 showed higher expression and activity in male compared to female in a hormonal-dependent manner (Ou-Yang et al. 2000; Relling et al. 1992). Higher levels of progesterone and estrogen, due to COCs use, luteal phase of the menstrual cycle or pregnancy, consistently led to a decrease in CYP1A2 activity (Balogh et al. 1995). In line with these results, in vitro investigations showed that estrogen can inhibit CYP1A2 activity (Eugster et al. 1993). For example, caffeine clearance is significantly faster in males compared to females (Nicolas et al. 2009; Rasmussen et al. 2002; Wong et al. 2021). As a substrate for CYP1A2, studies on warfarin indicate that women need less cumulative weekly dose compared to men (Garcia et al. 2005; Whitley et al. 2007). In addition, clearance of paracetamol (Abernethy et al. 1982a), olanzapine (Callaghan et al. 1999), and clozapine (Lane et al. 1999) has been shown to be higher in men compared to women.
- **CYP2E1** is responsible for the metabolism of over 70 compounds (Scandlyn et al. 2008). Few studies have documented that CYP2E1 activity was higher in males compared to females, resulting in 30% higher clearance of ethanol and chlorzoxazone (Lucas et al. 1995; Kim and O’Shea 1995). However, so far no clear evidence points towards a generally higher metabolism by CYP2E1 in men.
- **CES1** beyond oxidative metabolism, hydrolytic pathways contribute to drug activation, particularly for prodrugs. Female human livers exhibit higher carboxylesterase-1 (CES1) protein abundance and increased in vitro activation rates for several CES1 substrates, including oseltamivir (conversion to oseltamivir carboxylate) and dabigatran etexilate (conversion to dabigatran) (Shi et al. 2016a; Shi et al. 2016b). Consistently, women show higher systemic exposure to the active β-hydroxy acid metabolites of lovastatin and simvastatin, both CES1 substrates (Vree et al. 2003; Yang et al. 2014). By contrast, among patients receiving clopidogrel, women display higher on-treatment platelet reactivity, consistent with relative hyporesponsiveness; nevertheless, ischemic event rates are similar between sexes (Gasecka et al. 2023; Berger et al. 2009). A similar mechanism plausibly influences ACE-inhibitor prodrugs (e.g., enalapril to enalaprilat, (Liu et al. 2024; Her et al. 2021)), although clinical sex differences are less consistent and are frequently overshadowed by genetics (e.g., CES1 variants), renal function, and size.

#### Phase II reactions

Phase II metabolism involves conjugation reactions where functional groups introduced in phase I are linked to polar molecules, such as glucuronic acid, sulfate, or glutathione. These reactions, mediated by enzymes such as catechol-*O*-methyltransferase (COMT), thiopurine methyltransferase (TPMT), uridine diphosphate glucuronosyltransferase (UGT), and sulfotransferase (SULT), render drugs more water-soluble, facilitating their elimination through the kidneys or bile (Jancova et al. 2010). Sex differences in phase II metabolism are less pronounced than observed for phase I metabolism. For instance, TPMT levels and activity were higher in the liver of males (Szumlanski et al. 1992; Klemetsdal et al. 1992), requiring a higher dose of the cytostatic drug 6-mercaptopurine compared to female leukemia patients (Lennard et al. 1990). Moreover, cancer female patients show a dramatic lower clearance of fluorouracil (Milano et al. 1992; Port et al. 1991; Zalcberg et al. 1998) and doxorubicin (Dobbs et al. 1995) in parallel to changes in the activity of the hepatic dihydrouracil dehydrogenase and aldoketoreductase, respectively. In addition, the higher clearance of levodopa in male patients is linked to the increased activity of COMT, which is also responsible for the metabolism of the neurotransmitter dopamine, epinephrine, and norepinephrine (Boudikova et al. 1990; Floderus et al. 1981; Kumagai et al. 2014; Conti et al. 2022; Martinelli et al. 2003).

UGTs exhibit higher activity in men than in women (Relling et al. 1992; Bock et al. 1994). Consequently, drugs primarily eliminated through glucuronidation, such as oxazepam, may demonstrate a shorter half-life in men compared to women. This reduced UGT activity in women may lead to slower drug clearance and prolonged systemic exposure (Court et al. 2004). Moreover, the accelerated reactions in males fastened the elimination of propranolol (Schwartz 2003; Walle et al. 1989), labetalol (Johnson et al. 2000), and paracetamol (Bock et al. 1994; Allegaert et al. 2015). However, sex differences do not account for general difference in the metabolism of UGT glucuronosyltransferase substrates, including clofibric acid (Miners et al. 1984) or ibuprofen (Greenblatt et al. 1984). Minoxidil is converted to minoxidil-sulfate by follicular SULT1A1, the active vasodilatory species for hair growth. Response correlates with local SULT1A1 activity, which have been suggested to be higher in males. However, biological sex differences appear to reflect inter-individual enzyme activity more than systemic biological sex per se (Roberts et al. 2014; Pietrauszka and Bergler-Czop 2022; Jimenez-Cauhe et al. 2024). While nucleoside reverse-transcriptase inhibitors (NRTIs) are not classical hepatic conjugation, their sequential phosphorylation (mono/di/triphosphates) is the activation step. Multiple clinical studies show higher intracellular formation of the pharmacologically active triphosphates in females (approximately 2.3-fold for zidovudine-TP, 1.6-fold for lamivudine-TP, with higher carbovir-TP from abacavir) despite similar plasma parent drug levels (Moyle et al. 2009; Anderson et al. 2003).

The biological sex effect may vary with metabolizer status, sample size, or whether pharmacokinetic parameters are adjusted for body weight. Additionally, several factors can distort the interpretation of sex differences in drug metabolism, including genetic polymorphisms affecting enzymes like UGT, TPMT, and COMT, hormonal fluctuations, age-related changes, underlying health conditions, co-medication, and variations in study design (e.g., unbalanced sex ratios, inconsistent dosing strategies. Without careful control for these variables, it can be difficult to distinguish whether observed differences are genuinely due to biological sex or the result of confounding factors.

### Drug elimination

Drug elimination, the process of removing drugs and their metabolites from the body, primarily occurs through a collaboration between hepatic (described earlier in this chapter) and renal pathways. Without efficient elimination, drugs would continuously circulate, potentially leading to toxicity and prolonged pharmacological effects. Sex-specific differences in drug elimination arise due to physiological variations in renal function and hepatic clearance, influencing drug pharmacokinetics, efficacy and the related ADRs (see Table 4).

**Table 4:** Summary of observed biological sex differences in drug excretion

Parameter	Biological sex difference	Example	Reference
GFR	M > F	Digoxin, aminoglycosides, cephalosporins, fluoroquinolones methotrexate, fentanyl, torasemide	Schwartz 2003; Berg 2006; Silvaggio and Mattison 1994; Gross et al. 1992; Yukawa et al. 1992; Sweileh 2009; Frame et al. 1999; Barbhaiya et al. 1992; Reigner and Welker 1996; Godfrey et al. 1998; Nimmen et al. 2010; Kuip et al. 2017; Werner et al. 2010; Yukawa et al. 1997)
Renal Tubular Secretion	M > F	Probenecid, cimetidine	Nicolas et al. 2009; Schwartz 2003; Gaudry et al. 1993)

#### Glomerular filtration rate (GFR)

The kidneys serve as the primary organ for drug clearance, facilitating the elimination of both drug compounds and their metabolites through glomerular filtration, tubular secretion, and tubular reabsorption. Men generally demonstrate higher renal clearance compared to women, leading to faster drug elimination (Schwartz 2003; Gaudry et al. 1993; Berg 2006; Silvaggio and Mattison 1994). GF is the first step in renal drug elimination, which allows unbound drug molecules to pass through the kidney’s filtration barrier into the urine collecting tubules. Men found to exhibit higher GFR compared to women, even when adjusted for body weight and surface area (Berg 2006; Gross et al. 1992). This difference can be linked to greater renal blood flow, larger kidney mass, and generally higher blood flow in males, leading to enhanced filtration capacity (Cockcroft and Gault 1976). Thus, drugs that undergo primary elimination through glomerular filtration tend to be cleared more rapidly in men. Therefore, drug such as digoxin (Yukawa et al. 1992), aminoglycosides (e.g., gentamicin, amikacin, tobramycin) (Sweileh 2009), cephalosporins (Frame et al. 1999; Barbhaiya et al. 1992), and fluoroquinolones (Reigner and Welker 1996) often exhibit reduced clearance in women. Methotrexate demonstrates a 17% lower renal clearance in women compared to men, even after adjustments for body weight and creatinine clearance (Godfrey et al. 1998), and the urinary excretion of fentanyl was markedly decreased in women (Nimmen et al. 2010; Kuip et al. 2017). Furthermore, women on loop diuretics showed a higher rate ADRs compared to men (Werner et al. 2010). Generally, renal clearance estimations incorporate sex as a variable in creatinine clearance calculation. Since men produce more creatinine due to greater muscle mass, their baseline creatinine clearance values are higher than those observed in women (Cockcroft and Gault 1976). This has important implications for drug dosing, as formulas that fail to consider sex-specific renal function would lead to miscalculations, increasing the risk of drug accumulation and toxicity in female patients.

#### Renal tubular secretion and reabsorption

Beyond glomerular filtration, tubular secretion and reabsorption further modulate drug elimination. The secretion of organic cations (positively charged drugs) appears to be more efficient in men than in women, leading to faster drug elimination (Nicolas et al. 2009; Gaudry et al. 1993). Probenecid and cimetidine, which block active tubular secretion by targeting organic anion transporter (OAT)1 and 3, tend to have a greater impact in men, suggesting that male renal tubules have higher baseline transporter activity (Schwartz 2003; Gaudry et al. 1993). Conversely, renal tubular reabsorption shows less pronounced sex-specific variability, and sex hormonal influences on reabsorption remain incompletely understood.

### Biological sex-dependent pharmacokinetics of monoclonal antibodies

Therapeutic antibodies are eliminated predominantly by cellular uptake and lysosomal proteolysis via both nonspecific pinocytosis and target-mediated drug disposition, while renal filtration and hepatic metabolism are negligible (Ryman and Meibohm 2017; Centanni et al. 2019). The neonatal Fc receptor (FcRn) recycling modulates half-life (albumin as a covariate), and anti-drug antibodies (ADAs) can accelerate clearance by forming immune complexes (Roopenian and Akilesh 2007; Liu 2018). Across multiple population pharmacokinetic (PPK) analyses of anti–programmed cell death protein-1/ligand-1 (anti-PD-1/PD-L1) therapies, female biological sex is associated with lower clearance (22–27% lower than males) after accounting for body size and albumin (Ryman and Meibohm 2017; Centanni et al. 2019; Shang et al. 2022). The effect is reproducible but typically not dose-defining relative to other covariates (e.g., albumin, tumor burden, time varying clearance).

Multiple PPK models (including those for a licensed biosimilar) show higher clearance of bevacizumab in males (14–26%) and larger central volume, even after weight adjustment, with albumin inversely related to clearance (Han et al. 2016; Li et al. 2020). Among anti-TNF agents, males show higher ADAs and lower plasma concentration of, whereas adalimumab shows no biological sex differences (Fasanmade et al. 2009). For rituximab, men have faster clearance (e.g., ~ 12.7 vs 8.2 mL/h) and shorter half-life than women, which was linked to worse outcomes in older men with diffuse large B-cell lymphoma (DLBCL) (Pfreundschuh et al. 2014; Muller et al. 2012). However, trastuzumab deruxtecan and several other mAbs, biological sex can enter models as a statistically significant covariate, but its impact on exposure is typically < 20% and not dose-defining relative to body weight, albumin, tumor burden, and time-varying disease effects (Yin et al. 2021).

## Other sex-specific influences on pharmacokinetics in women

### Menstrual cycle and oral contraceptives

The variations in hormone levels and the physiological changes throughout the menstrual cycle could potentially have a significant effect on the pharmacokinetics of drugs. However, there is no clear evidence suggesting a menstrual cycle influence on drug absorption and distribution. Some studies indicate that gastrointestinal transit time and stool wet and dry weights vary during the menstrual cycle. It appears that absorption in the small intestine may be increased during the luteal phase due to a prolonged gastrointestinal transit time. It has been suggested that relatively high levels of progesterone, which can promote smooth muscle relaxation, may be responsible for this effect (Sweeting 1992; McBurney 1991). Additionally, the absorption of alcohol (Jones and Jones 1976) and salicylates (Miaskiewicz et al. 1982) appears to be slower at mid-cycle while theophylline concentration was at the peak at mid-cycle compared to other stages in the menstrual cycle (Bruguerolle et al. 1990).

Throughout the menstrual cycle, fluctuations in sex hormone concentrations may influence hepatic enzyme activity. Progesterone has been shown to both inhibit and induce hepatic enzyme activity at various stages of the cycle (Lai et al. 2020). For instance, the incidence of ibutilide-induced long QT syndrome is higher in females compared to males; additionally, the incidence has been shown to be higher during the ovulatory phase than in the luteal phase (Rodriguez et al. 2001; Hreiche et al. 2009; Johnson et al. 1997). The clearance rates of caffeine and theophylline are higher in the early follicular phase and decrease during the mid-luteal phase (Buchanan et al. 2009). These differences in the plasma concentration during different phases of the menstrual cycle can be translated to clinical symptoms as documented in some asthmatic women who experience worsened symptoms before or during menstruation due to higher theophylline clearance (Miller et al. 2007; Nagata et al. 1997). Females exhibit a greater sensitivity to opioids, possibly due to the effects of female hormones on the density of opioid receptors or changes in GABA receptors, leading to a higher incidence of ADRs (Ueno 2009; Frye and Duncan 1994). Recent studies also highlight the potential effect of estrogen, as a substrate for CYP3A4 and CYP1A2, on the antidepressant metabolism during the late luteal phase of the menstrual cycle or with estrogen replacement therapy (Bigos et al. 2009).

Oral contraceptives (OCs) counteract the metabolism of numerous drugs through several mechanisms. Predominantly, the ethynyl-containing steroids in most OCs act as inhibitors of cytochromes P450 (Guengerich 1990; Ortiz Montellano and Kunze 1980), specifically targeting CYP3A4, CYP1A2, CYP2B6, CYP2C19, and CYP2C9 among other isozymes. This interaction reduces the hepatic metabolism of various drugs such as cyclosporin, corticosteroids, theophylline, carisoprodol, selegiline, phenytoin imipramine, amitriptyline, prednisolone, and diazepam, decreasing their clearance by up to 50% (Guengerich 1990; Teichmann 1990; Back and Orme 1990; Meffin et al. 1984; Abernethy et al. 1982b; Abernethy et al. 1984; Edelbroek et al. 1987; Sandberg et al. 2004; Granfors et al. 2005; Matthaei et al. 2016; Abernethy and Todd 1985; Gu et al. 1992; Tornatore et al. 1982; Tamminga et al. 1999; Laine et al. 1999; Bramness et al. 2005). For instance, a clinical study showed that ethinylestradiol increased the area under the curve (AUC) of omeprazole by 38%, though this interaction is unlikely to be clinically significant due to omeprazole’s wide therapeutic index (Palovaara et al. 2003).

OCs are known to increase the glucuronidation clearance of drugs such as clofibrate (Miners et al. 1984; Liu et al. 1991), propranolol (Walle et al. 1996), phenprocoumon (Monig et al. 1990), diflunisal (Herman et al. 1994), and lamotrigine (Sabers et al. 2001). Additionally, OCs elevate the levels of glucuronosyl transferases, which enhances the conjugation of drugs like paracetamol, which has been shown to increase by 48% in OC users compared to non-users (Miners et al. 1983; Mitchell et al. 1983). The specific mechanism behind this increase in enzyme levels remains unclear.

### Menopause

As menopause occurs, there is a significant reduction in circulating estrogen levels, decreasing to about 10% of pre-menopausal levels. During this phase, the conversion of androgens to estrogen primarily occurs in adipose tissue and skin, facilitated by the enzyme aromatase, encoded by CYP19A1. This shift leads to changes in drug metabolizing enzymes, including a reduction in CYP3A4 activity in the intestine by about 20% (Paine et al. 2005). Such changes can affect the metabolism of medications that utilize this pathway, affecting their efficacy during and after the menopausal transition. Additionally, the use of exogenous hormones to alleviate menopausal symptoms such as hot flashes, night sweats, vaginal dryness, and sleep disturbances could modify the metabolism of particular drugs. Despite these interactions, clinical data on the exact impact of menopause and its physiological changes on drug pharmacokinetics remain inconclusive. To examine menopause-related alterations in intestinal or hepatic CYP3A4 activity, several studies compared the pharmacokinetics of midazolam, erythromycin, and prednisolone clearance in pre- and postmenopausal women and found no significant differences in drug metabolism according to menopausal status (Harris et al. 1996).

### Pregnancy

During early pregnancy, nausea and vomiting may diminish bioavailability of drugs following oral administration. Concurrently, a decrease in gastric acid production coupled with an increase in mucus secretion elevates gastric pH. This shift may alter the absorption of many drugs, by for example increasing the ionization of weak acids like aspirin, which reduces their absorption. Moreover, this may allow weak bases like caffeine to readily diffuse due to their primarily unionized state. However, confirmatory evidence for these assumptions remains elusive (Vasicka et al. 1957; Waldum et al. 1980; Hong et al. 2005; Lawson et al. 2025). Cardiovascular manifestations during pregnancy include a significant increase in cardiac output, which commences early in pregnancy and stabilizes at approximately 7 L per minute by 16 weeks of gestation, maintaining this elevated level until delivery (Qasqas et al. 2004). Additionally, there is an observed increase in stroke volume beginning at 4 months of gestation, with maternal heart rate progressively increasing to about 90 beats per minute at rest by the third trimester (Pirani et al. 1973). Concurrent with these cardiovascular changes, pregnancy is marked by a significant increase in plasma volume (around 3.5 L) at the end of gestation (Qasqas et al. 2004). This expansion in all fluid compartments may increase the volume of distribution for hydrophilic drugs, leading to sub-therapeutic plasma concentrations. Moreover, the maternal fat composition increases by approximately 4 kg, which may enhance the volume of distribution for lipophilic drugs (Lederman et al. 1997). However, data assessing the role of adipose tissue mass in altering drug disposition during pregnancy are limited. Additionally, the free (unbound) concentration of certain drugs can decrease during pregnancy due to a notable drop of albumin and alpha-1-acid glycoprotein concentration (Murphy et al. 2002; Cheung et al. 1989; Hayashi et al. 2002). These changes can be clinically relevant for drugs such as phenytoin and tacrolimus, where the effect is closely related to unbound drug in the plasma. During pregnancy, both drugs show an increased unbound fraction and enhanced clearance (Hebert et al. 2013; Perucca and Crema 1982).

The enzymatic activity of several CYP450 isoforms, such as CYP3A4, CYP2A6, CYP2D6, and CYP2C9, is increased during pregnancy, influencing the metabolism of drugs like midazolam, phenytoin, glyburide, nifedipine, and nelfinavir (Hebert et al. 2008; Hirt et al. 2006; Villani et al. 2006; Tracy et al. 2005; Carter et al. 1986; Quinney et al. 2012; Dickinson et al. 1989; Hebert et al. 2009). Conversely, CYP1A2 and CYP2C19 show a gradual decrease in activity as gestation progresses, though the clinical implications of these changes on drug therapy remain uncertain (Brazier et al. 1983; Tsutsumi et al. 2001; Grosso and Bracken 2005). The activity of phase II enzymes, like UGTs, may also change during pregnancy. UGT1A4 activity undergoes a twofold increase in the first and second trimesters and a threefold increase in the third trimester (Haan et al. 2004). This enhancement leads to lower concentrations of UGT1A4 substrates like lamotrigine, directly affecting seizure control in the absence of appropriate dose adjustments (Haan et al. 2004). These changes in drug metabolism require consideration of drug dosing, especially for medications with a narrow therapeutic window. On the other hand, adjustments in drug dosages may also be required in the postpartum period to avoid increased toxicity when pregnancy-related metabolic changes resolve.

Furthermore, GFR is elevated by 50% by the first trimester and continues to increase until the end of pregnancy (Davison and Dunlop 1980). Thus, the clearance of renal-eliminated drugs is expected to be in line with the changes in GFR during pregnancy. For instance, antibiotics like cefazolin and ampicillin demonstrate increased renal elimination, and clindamycin shows increased clearance during pregnancy (Allegaert et al. 2009; Muller et al. 2010; Chamberlain et al. 1993). Despite this uniform increase in GFR, variations in renal tubular transport (either secretion or reabsorption) can produce different effects on renally cleared drugs (Muller et al. 2010). The clearance of lithium for example is doubled during the third trimester compared to pre-pregnancy levels. However, the clearance of digoxin, which is largely eliminated via the kidneys, only increases by 20–30% during the third trimester relative to the postpartum period (Hebert et al. 2008; Syme et al. 2004; Schou et al. 1973). Additionally, atenolol renal clearance increases by 36–38% in the 2nd–3rd trimesters, while half-life decreases by 11–12% (Hebert et al. 2005). These discrepancies highlight the importance of considering further research on gestational changes in renal drug elimination. Table 5 summarizes the pharmacokinetic differences in pregnancy.

**Table 5:** Drug pharmacokinetics differences in pregnancy

Parameter	Difference	Example	Reference
Drug absorption and distribution			
Gastric pH and mucus secretion	Pregnant > non-pregnant	-	Vasicka et al. 1957; Waldum et al. 1980; Hong et al. 2005; Lawson et al. 2025)
CO, stroke volume and plasma volume	Pregnant > non-pregnant	Probenecid, cimetidine	Qasqas et al. 2004; Pirani et al. 1973)
Body fat composition	Pregnant > non-pregnant	-	Lederman et al. 1997)
Plasma protein binding	Pregnant < non-pregnant	Phenytoin, tacrolimus	Hebert et al. 2013; Perucca and Crema 1982)
Drug metabolism			
CYP3A4, CYP2A6, CYP2D6, and CYP2C9	Pregnancy > F	Glyburide, nifedipine, nelfinavir, midazolam, phenytoin	Hebert et al. 2008; Hirt et al. 2006; Villani et al. 2006; Tracy et al. 2005; Carter et al. 1986; Quinney et al. 2012; Dickinson et al. 1989; Hebert et al. 2009)
CYP1A2 and CYP2C19	Pregnancy < F	-	Brazier et al. 1983; Tsutsumi et al. 2001; Grosso and Bracken 2005)
UGTs (UGT1A4)	Pregnancy > F	Lamotrigine	Haan et al. 2004)
Drug elimination			
GFR	Pregnancy > F	Cefazolin, ampicillin clindamycin, lithium, digoxin and atenolol	Hebert et al. 2008; Davison and Dunlop 1980; Allegaert et al. 2009; Muller et al. 2010; Chamberlain et al. 1993; Syme et al. 2004; Schou et al. 1973; Hebert et al. 2005; Philipson 1977; Lopes van Balen et al. 2019)

## Data Availability

All source data for this work (or generated in this study) are available upon reasonable request.
